# Foot-and-mouth disease virus activates glycolysis and hijacks HK2 to inhibit innate immunity and promote viral replication

**DOI:** 10.1186/s13567-025-01497-w

**Published:** 2025-04-01

**Authors:** Wenxian Chen, Xinyan Wang, Xiaowen Li, Weijun Wang, Yaoyao Huang, Yuwei Qin, Pengfei Liu, Keke Wu, Bingke Li, Yintao He, Sen Zeng, Lin Yi, Lianxiang Wang, Mingqiu Zhao, Hongxing Ding, Shuangqi Fan, Zhaoyao Li, Jinding Chen

**Affiliations:** 1 College of Veterinary Medicine, South China Agricultural University, Guangzhou, China; 2 State Key Laboratory for Animal Disease Control and Prevention, College of Veterinary Medicine, Lanzhou Veterinary Research Institute, Chinese Academy of Agricultural Sciences, Lanzhou University, Lanzhou, 730000 China; 3 Wen’s Foodstuffs Group Co., Ltd., Yunfu, Xinxing China

**Keywords:** Foot-and-mouth disease virus, glycolysis, hexokinase 2, replication, interferon regulatory factor

## Abstract

Foot-and-mouth disease (FMD) severely restricts the healthy development of global animal husbandry, and the unclear pathogenic mechanism of FMD virus (FMDV) leads to difficulty in preventing and purifying FMD. Glycolytic remodelling is considered one of the hallmarks of viral infection, providing energy and precursors for viral assembly and replication. In this work, the interaction and mechanism between FMDV and glycolysis were explored from the perspective of immune metabolism. We found that FMDV infection increased the extracellular acidification rate, lactic acid accumulation, and HK2 level. In addition, during FMDV infection, HK2 enhances glycolytic activity and mediates autophagic degradation of IRF3/7 to antagonize the innate immune response, thereby promoting viral replication. Our findings provide evidence that FMDV is closely correlated with host metabolism, increasing the understanding that glycolysis and HK2 facilitate virus infection, and provide new ideas for further elucidating the pathogenic mechanism of FMDV.

## Introduction

Foot-and-mouth disease (FMD) is a highly contagious disease caused by foot-and-mouth disease virus (FMDV) infection, which seriously endangers the development of animal husbandry. At present, the pathogenic mechanism of FMDV is unknown, which severely hinders its prevention and control. Therefore, it is crucial to identify the key host pathways and target molecules involved in FMDV infection, which is beneficial for further understanding the pathogenic mechanism of the virus.

The virus is an intracellular parasite whose survival and reproduction depend on the metabolic system of the host cell. Viruses are able to utilize various metabolic pathways in the host to meet the bioenergy and biosynthetic conditions required for the production of progeny viruses [1–3]. Glycolysis is the process of converting glucose into pyruvate in the cellular solute through a series of enzymatic reactions, thereby producing the energy source [4]. Studies have shown that viruses can manipulate and reshape glycolytic pathways to maintain their own proliferation, and glycolytic reprogramming is considered one of the hallmarks of viral infection [5–7]. Senecavirus A (SVA) can induce glycolysis and increase lactate production to promote viral replication [8]. Dengue virus (DENV) requires glycolysis for optimal replication, and the NS1 protein can increase glycolytic flux [9, 10].

Hexokinase 2 (HK2) is the first rate-limiting enzyme of glycolysis and is involved in glucose phosphorylation [11]. HK2 is regarded as the key molecule that adjusts energy metabolism and cell fate in various cells [12]. Recent research has revealed the important role of HK2 in tumors and cancers. For example, in breast cancer studies, OTUB1 enhances the transcriptional activity of MYC and induces the expression of HK2, thus promoting aerobic glycolysis [13]. HK2 accelerated the autophagy-dependent degradation of AIMP2 to attenuate apoptosis and promote proliferation in HCC cell lines [14]. Viruses also use HK2 to influence host metabolism, thereby creating a microenvironment conducive to viral replication. HK2 is involved in the development of autophagy during CSFV infection to regulate innate immunity [15]. DENV infection induced autophagy in vivo and in vitro and promoted viral replication by activating glycolysis through autophagy [16]. In addition, HK2 protects cardiomyocytes by promoting autophagy in response to glucose deprivation, and HK2 acts as a molecular transition switch from glycolysis to autophagy to ensure cell energy homeostasis under starvation conditions [17]. Taken together, these studies reveal a close correlation between glycolysis and autophagy, which has important implications for the regulation of cell metabolism.

FMDV has been reported to induce autophagy and negatively regulate innate immunity via autophagy for viral replication [18, 19]. In this study, we focused on analysing the mutual regulatory relationship between FMDV and glycolytic metabolism and revealed that FMDV infection activated the glycolytic process and hijacked HK2 to antagonize the innate immune response, thereby promoting the FMDV infection process. HK2 further increased autophagy flux during FMDV infection and interacted with IRF3/7 to mediate the autophagy‒lysosome-dependent degradation of IRF3/IRF7. This study reveals that FMDV regulates metabolism to evade the immune response and helps us better understand the regulatory relationships among viral infection, metabolism and autophagy.

## Materials and methods

### Reagents and antibodies

The chemical reagents used in this study were as follows: 2-deoxy-D-glucose (2-DG) (Macklin, D807272), rapamycin (MedChemExpress, HY-10219), bafilomycin A1 (MedChemExpress, HY-100558), chloroquine (CQ) (MedChemExpress, HY-17589A), Dulbecco’s modified Eagle’s medium (DMEM) (Thermo Fisher Scientific, 11,995,040), Opti-MEM I reduced serum medium (Thermo Fisher Scientific, 11,058,021), fetal bovine serum (FBS) (Thermo Fisher Scientific, 10,099), and Lipofectamine 3000 transfection reagent (Thermo Fisher Scientific, L3000015).

The primary antibodies used in this study were as follows: rabbit polyclonal anti-HK2 (Proteintech, 2A11C3), rabbit polyclonal anti-FMDV VP1 (Bioss, bs-41049R), mouse monoclonal anti-p62 (Cell Signaling Technology, 8025), rabbit polyclonal anti-LC3B (Cell Signaling Technology, 2775), and mouse monoclonal anti-β-actin (Abmart, TP70573) antibodies.

### Cell culture and viral infection

The swine kidney cell lines PK-15 and HEK293T were preserved in our laboratory. The cells were grown in DMEM containing 10% FBS and cultured at 37 °C in a 5% CO_2_ incubator. The FMDV type O strain O/BY/CHA/2010 was propagated in BHK-21 cells via standard virology techniques, and the supernatants of the infected cells were clarified and stored at −80 °C. The virus titres were determined with 50% tissue culture infective dose (TCID_50_) assays.

### Western blot analysis

Western blot analyses used in this research were performed as previously described [20, 21]. The cell samples were sequentially washed with cold PBS twice, incubated with cell lysis buffer (Beyotime Biotechnology, P0013B) containing 1 mM PMSF (Beyotime Biotechnology, ST506) on ice for 20 min and centrifuged (13 000 *g*/min for 15 min at 4 °C). The supernatant was collected. The protein content of the supernatant was measured with a BCA protein assay kit (Beyotime Biotechnology, P0012) and then boiled for 10 min in 5 × SDS‒PAGE sample loading buffer (Beyotime Biotechnology, P0015L). After separation via 12.5% SDS‒PAGE, the proteins were transferred onto a polyvinylidene difluoride (PVDF) membrane and blocked with PBS containing 5% non-fat milk powder and 0.1% Tween 20 at 37 °C for 1 h, followed by an overnight incubation at 4 °C with primary antibodies. Afterwards, the PVDF membranes were incubated with the corresponding secondary antibodies conjugated to HRP at 37 °C for 1 h. The immunolabelled protein complexes were visualized via an enhanced chemiluminescence (ECL) Plus Kit (Beyotime Biotechnology, P0018) via the CanoScan LiDE 100 scanner system (Canon).

### Quantitative real-time RT–PCR (qRT‒PCR)

The quantitative real-time RT–PCR assay used in this research was performed as previously described [20]. Total RNA from different cell samples was extracted via a total RNA Kit I (Omega, R6834-02). cDNA synthesis was performed with HiScript II Q RT SuperMix (Vazyme, R223-01). The qRT‒PCR assay was executed with ChamQ Universal SYBR qPCR Master Mix (Vazyme, Q711-02) utilizing an iQ5 iCycler detection system (Bio-Rad, 1,855,200). Relative mRNA expression was determined using the 2^−ΔΔCT^ method and normalized to that of the housekeeping gene β-actin.

### Confocal immunofluorescence microscopy

The cells were cultured and placed in 35 mm Petri dishes (NEST, GBD-35–20) with a glass bottom. After the corresponding transfection or infection treatment, the cells in the dish were treated in the following steps: washing with PBS 3 times, fixing with 4% paraformaldehyde (Sigma‒Aldrich, P6148) for 30 min at room temperature, permeating with 0.1% Triton X-100 (Sigma‒Aldrich, T8787) for 10 min, and staining the nuclei with DAPI (Beyotime Biotechnology, C1002). The fluorescence signals were observed with a TCS SP2 confocal fluorescence microscope (Leica TCS SP8).

### Statistical analysis

The data are shown as the mean ± standard deviation (SD) and were analysed by t tests (and nonparametric tests), one-way ANOVA or two-way ANOVA tests with GraphPad Prism 9. *P* values of less than 0.05 were considered statistically significant.

## Results

### FMDV infection induced glycolytic metabolism and enhanced the expression of HK2

**Fig. 1:**
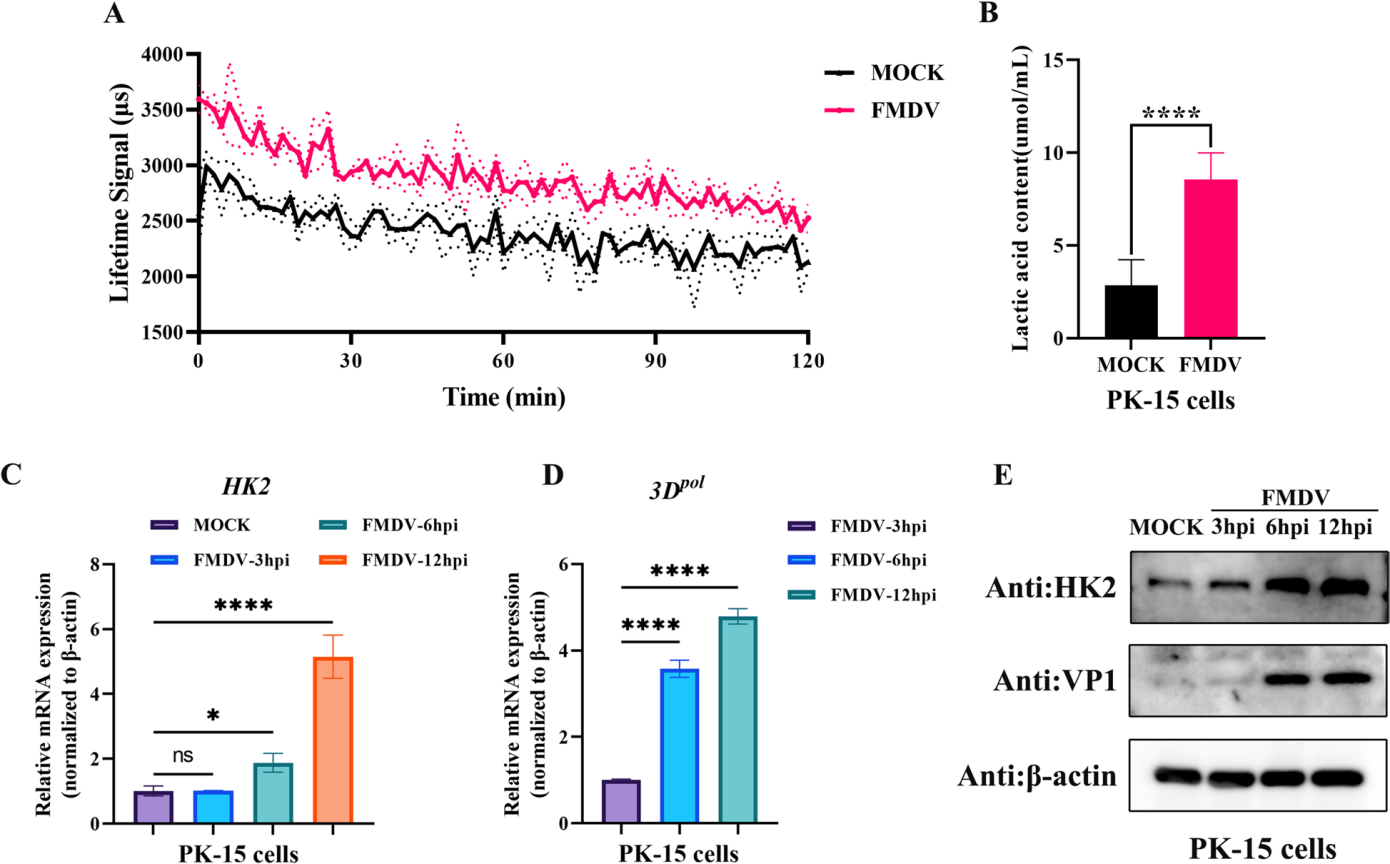
**FMDV triggered glycolysis and HK2 expression**. **A** PK-15 cells were infected with 0.5 MOI FMDV for 12 h, and the ECAR was monitored with a fluorescence microplate reader. **B** ELISA was used to measure the concentration of lactic acid. The error bars indicate the mean (± SD) of 3 independent experiments. *, *P* < 0.05; **, *P* < 0.01; and ***, *P* < 0.001 (t tests). (C and D) qRT‒PCR analysis of the relative mRNA expression of HK2 and 3D^pol^ in FMDV-infected PK-15 cells at different times (0 h, 3 h, 6 h and 12 h). The error bars indicate the mean (± SD) of 3 independent experiments. *, *P* < 0.05; **, *P* < 0.01; and ***, *P* < 0.001 (one-way ANOVA). (D) HK2 and FMDV VP1 protein expression in FMDV-infected PK-15 cells at different times, as determined by western blotting.

Viruses can remodel the host metabolic environment, including through glycolysis, to provide energy and biosynthetic precursors for their own replication, assembly and release [22–24]. To clarify the effect of FMDV infection on cellular glycolytic metabolism, PK-15 cells were infected with FMDV for 12 h, and the extracellular acidification rate (ECAR) was detected since it is an important index for evaluating the energy metabolism and growth state of cells and can provide rich information concerning glycolytic activity. The results revealed that the ECAR in FMDV-infected cells was greater than that in control cells (Figure 1A). Lactic acid is the final product of glycolysis. We further measured the change in lactic acid content in FMDV-infected and uninfected cells. As shown in Figure 1B, FMDV infection increased the lactic acid content in PK-15 cells. These results revealed that FMDV infection promoted glycolysis in PK-15 cells.

Hexokinase 2 (HK2) is the key metabolic enzyme in the first step of the glycolytic pathway [12]. Studies have shown that viruses such as DENV, HBV and CSFV can regulate host metabolism via HK2 to assist in their own replication [10, 15, 25]. We further explored whether FMDV affects the glycolysis metabolic enzyme HK2. PK-15 cells were infected with FMDV for different durations (0 h, 3 h, 6 h and 12 h). The mRNA levels of the HK2 and FMDV 3D^pol^ genes were measured by qRT‒PCR, and the protein expression of HK2 and FMDV VP1 was assessed via western blotting. The results indicated that the mRNA and protein levels of HK2 in PK-15 cells increased with increasing duration of FMDV infection (Figures 1C–E).

### The glycolytic metabolic enzyme HK2 promoted FMDV replication

**Fig. 2:**
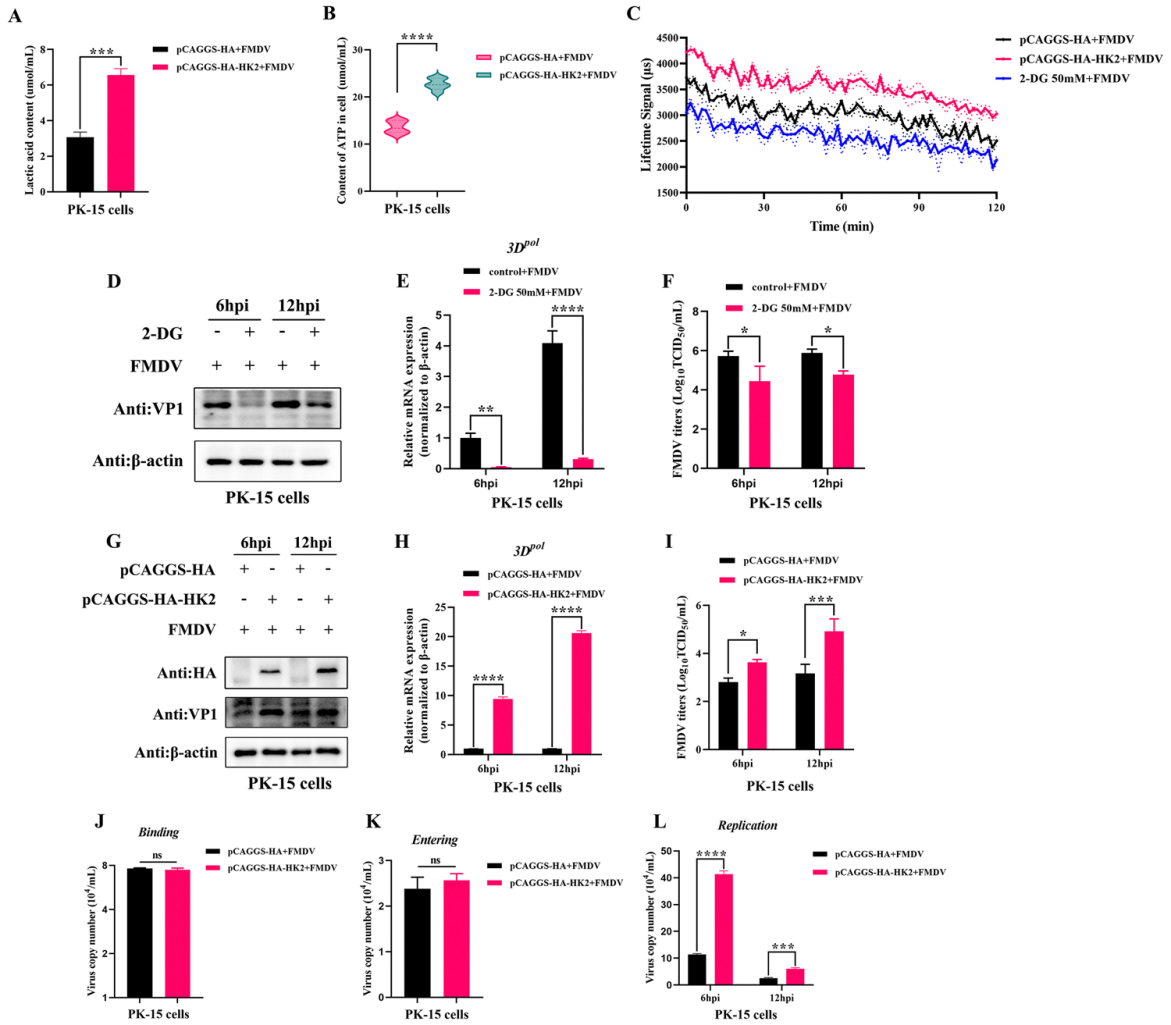
**HK2 enhanced glycolysis and FMDV replication in PK-15 cells**. **A** PK-15 cells overexpressing the pCAGGS-HA or pCAGGS-HA-HK2 plasmid were infected with FMDV for 12 h, and the lactic acid content in the cells was measured by ELISA. The error bars indicate the mean (± SD) of 3 independent experiments. *, *P* < 0.05; **, *P* < 0.01; and ***, *P* < 0.001 (t tests). **B** ELISA was used to measure the concentrations of ATP in FMDV-infected PK-15 cells overexpressing the pCAGGS-HA or pCAGGS-HA-HK2 plasmid. The error bars indicate the mean (± SD) of 3 independent experiments. *, *P* < 0.05; **, *P* < 0.01; and ***, *P* < 0.001 (t tests). **C** PK-15 cells pretreated with the pCAGGS-HA plasmid, pCAGGS-HA-HK2 plasmid or 2-DG (50 mmol/L) were infected with FMDV for 12 h, and the ECAR levels were monitored with a fluorescence microplate reader. **D**–**F** PK-15 cells were treated with 50 mmol/L 2-DG for 4 h and then infected with FMDV for 6 h and 12 h. FMDV VP1 protein expression (D), FMDV 3D^pol^ mRNA expression (**E**) and virus titres (F) were measured by western blot, qRT‒PCR and TCID_50_ survey, respectively. The error bars indicate the mean (± SD) of 3 independent experiments. *, *P* < 0.05; **, *P* < 0.01; and ***, *P* < 0.001 (two-way ANOVA). (G-I) PK-15 cells were infected with the pCAGGS-HA-HK2 plasmid for 24 h and then infected with FMDV for 6 h and 12 h. FMDV VP1 protein expression (**G**), FMDV 3D^pol^ mRNA expression (**H**) and virus titres (**I**) were measured by western blot, qRT‒PCR and TCID_50_ survey, respectively. The error bars indicate the mean (± SD) of 3 independent experiments. *, *P* < 0.05; **, *P* < 0.01; and ***, *P* < 0.001 (two-way ANOVA). **J** After the pCAGGS-HA plasmid or pCAGGS-HA-HK2 plasmid was overexpressed for 24 h, PK-15 cells were infected with FMDV for 1 h at 4 ℃, and then, the number of virus copies was determined via qRT‒PCR. (**K**) After the pCAGGS-HA plasmid or pCAGGS-HA-HK2 plasmid was overexpressed for 24 h, PK-15 cells were infected with FMDV for 1 h in 4 ℃, and then washed with PBS, and cultured with 2% FBS DMEM at 37 ℃ for 30 min. The number of virus copies was detected via qRT‒PCR. **L** After the pCAGGS-HA plasmid or pCAGGS-HA-HK2 plasmid was overexpressed for 24 h, PK-15 cells were infected with FMDV for 6 h and 12 h, and the number of virus copies was detected via qRT‒PCR. (J-L) Error bars indicate the mean (± SD) of 3 independent experiments. *, *P* < 0.05; **, *P* < 0.01; and ***, *P* < 0.001 (t tests).

**Fig. 3:**
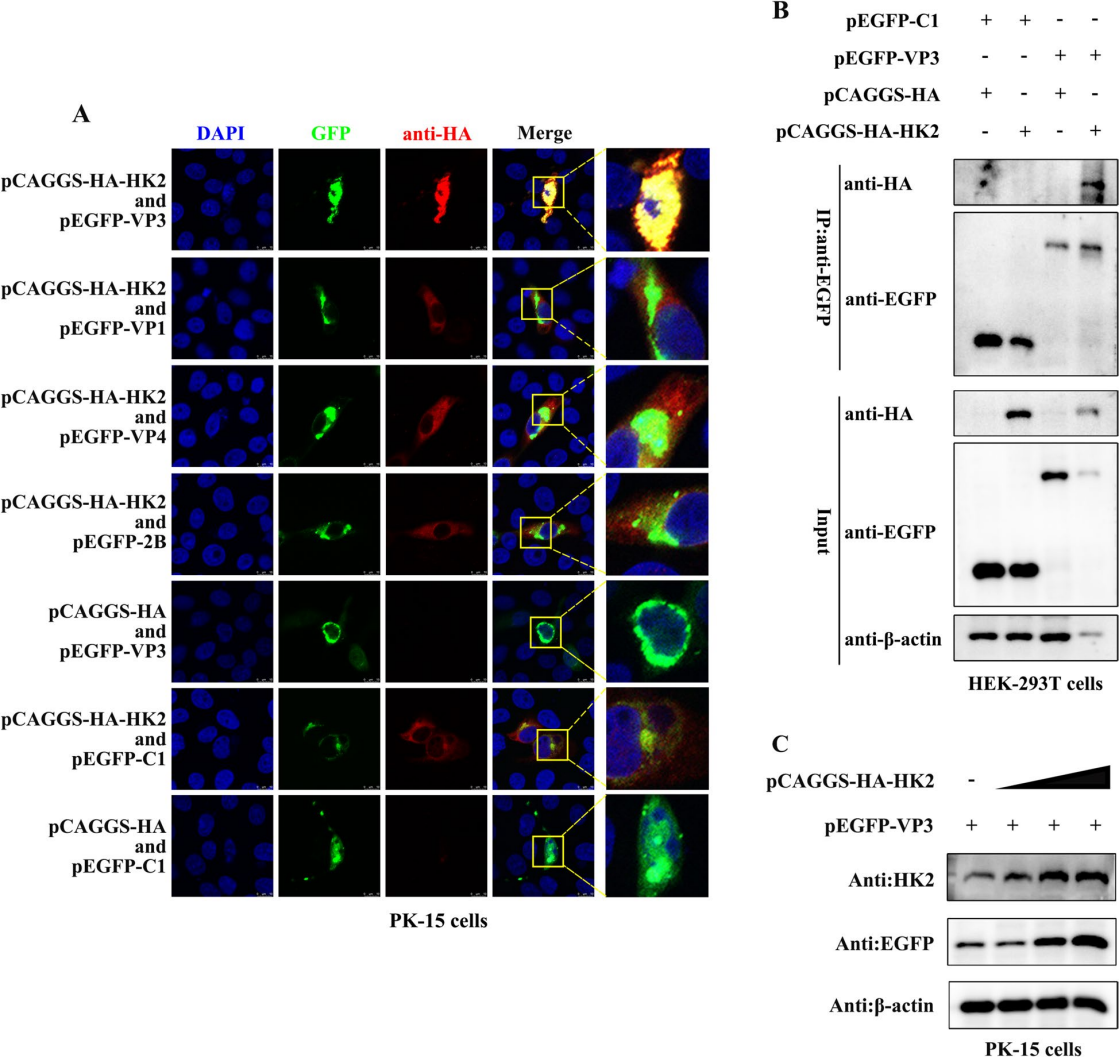
**Interaction of HK2 with the FMDV VP3 protein**. **A** PK-15 cells were co-transfected with pCAGGS-HA-HK2 and different FMDV protein expression plasmids (pEGFP-VP1, pEGFP-VP3, pEGFP-VP4 and pEGFP-2B) for 24 h, fixed, stained with DAPI (blue) and antibodies against GFP (green) and HA (red), and then examined by confocal microscopy. **B** pCAGGS-HA-HK2 and pEGFP-VP3 were transfected into 293T cells together. At 24 h after transfection, the cell lysates were immunoprecipitated with anti-GFP agarose beads. The bound proteins were eluted and detected by western blotting with anti-HA and anti-FLAG monoclonal antibodies. **C** PK-15 cells were transfected with 0 μg, 1 μg, 2 μg or 3 μg of pCAGGS-HA-HK2 together with pEGFP-VP3 for 24 h, and the expression of HK2 and VP3 was tested by western blotting.

HK2 plays a vital role in glycolysis. The contents of lactic acid, ATP and ECAR in FMDV-infected PK-15 cells overexpressing HK2 were measured to identify the importance of HK2 in the glycolysis process induced by FMDV infection. The results revealed that both the levels of lactic acid (Figure 2A) and ATP (Figure 2B) in FMDV-infected PK-15 cells overexpressing HK2 were greater than those in the control group. Furthermore, in the FMDV infection state, the ECAR in PK-15 cells decreased after 2-DG treatment but significantly increased after HK2 overexpression (Figure 2C). 2-DG is a commonly used competitive inhibitor of hexokinase and can antagonize glycolysis pathways [26]. We further verified the effect of glycolysis on FMDV replication. After 4 h of pretreatment of PK-15 cells with or without 2-DG, the cells were infected with FMDV at different times (6 h and 12 h), and FMDV replication in the cells was monitored. The results revealed that FMDV VP1 protein expression (Figure 2D) and FMDV 3D^pol^ mRNA expression (Figure 2E) in PK-15 cells were significantly decreased after 2-DG treatment, and the virus titre in FMDV-infected cells was also obviously lower than that in the 2-DG-untreated group (Figure 2F). The effect of HK2 on FMDV replication was further investigated, and HK2 overexpression increased FMDV VP1 protein expression (Figure 2G) and 3D^pol^ (Figure 2H) gene levels, as well as virus titres (Fig. 2I). These results indicate that HK2 is a key molecule in the regulation of FMDV proliferation via glycolysis. Next, to explore how HK2 affects the proliferation of FMDV, we tested the ability of FMDV to adsorb, invade and replicate in PK-15 cells overexpressing HK2. The results showed that HK2 did not affect the absorption or invasion of FMDV in PK-15 cells (Figures 2J-K). However, the replication process of FMDV was influenced by HK2 (Figure 2L).

On the basis of the above studies, there is mutual control between FMDV and the glycolytic metabolic enzyme HK2. FMDV proteins that interact with HK2 were further screened. The pCAGGS-HA-HK2 plasmid was transfected into PK-15 cells with pEGFP-VP1, pEGFP-VP3, pEGFP-VP4 or pEGFP-2B for 24 h, and the localization of the proteins was observed via confocal immunofluorescence microscopy. As shown in Figure 3A, pCAGGS-HA-HK2 colocalized with only pEGFP-VP3. Furthermore, a coimmunoprecipitation (co-IP) assay was performed by western blotting, and the results revealed that pCAGGS-HA-HK2 coprecipitated with pEGFP-VP3 in 293T cells (Figure 3B). In conclusion, these results indicate that FMDV VP3 interacts with the host protein HK2. In addition, we analysed the effect of HK2 on the VP3 protein and discovered that HK2 enhanced the expression of the FMDV VP3 protein (Figure 3C).

### HK2 promoted FMDV replication by inhibiting the expression of interferon-associated cytokines through autophagy

**Fig. 4:**
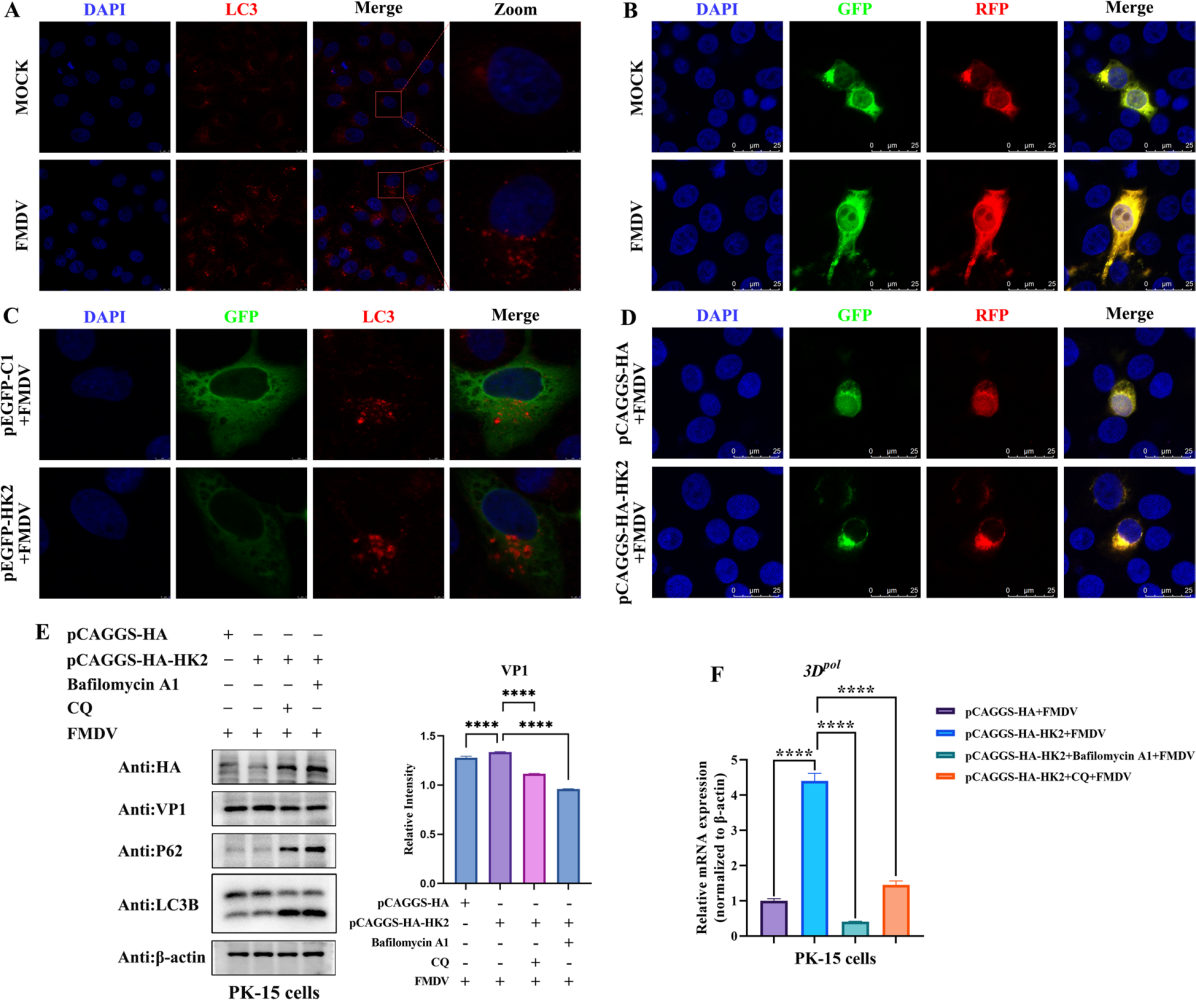
**HK2 enhances FMDV replication through autophagy**. **A** PK-15 cells were infected with FMDV for 12 h, and the number of endogenous LC3 puncta was determined by confocal microscopy. **B** PK-15 cells were transfected with dual fluorescence-tagged mRFP-eGFP-LC3 for 24 h, followed by FMDV infection for 12 h. Changes in the fluorescence of mRFP-eGFP-LC3 were observed by confocal microscopy. **C** PK-15 cells were transfected with pEGFP-C1 or pEGFP-HK2 for 24 h, followed by FMDV infection for 12 h. The number of endogenous LC3 puncta was determined by confocal microscopy. **D** PK-15 cells were co-transfected with dual fluorescence-tagged mRFP-eGFP-LC3 and pCAGGS-HA or with dual fluorescence-tagged mRFP-eGFP-LC3 and pCAGGS-HA-HK2 for 24 h, followed by FMDV infection for 12 h. Changes in the fluorescence of mRFP-eGFP-LC3 were observed by confocal microscopy. **E** and **F** PK-15 cells overexpressing pCAGGS-HA or pCAGGS-HA-HK2 were treated or not treated with bafilomycin A1 or CQ for 4 h and then infected with FMDV for 12 h. Western blotting was used to evaluate the protein expression of HA, VP1, P62, LC3B and β-actin (E), and FMDV 3D^pol^ mRNA levels were measured by qRT‒PCR (**F**).

Autophagy is a catabolic process necessary to maintain cell homeostasis and plays a crucial role in resisting the invasion of pathogenic microorganisms [27]. Interestingly, a variety of viruses have evolved mechanisms to inhibit, circumvent, or manipulate autophagy to promote their survival. Recent studies have shown that FMDV induces autophagy to antagonize antiviral interferon responses for self-replication [28]. We first observed the number of endogenous LC3 dot clusters induced by FMDV infection. As shown in Figure 4A, the number of endogenous LC3 puncta increased in FMDV-infected PK-15 cells. Next, dual fluorescence-tagged mRFP-eGFP-LC3 was utilized to detect FMDV-induced autophagy since it can be used to reflect the level of autophagy. We found that PK-15 cells with mRFP-EGFP-LC3 expression presented more red fluorescence after being infected with FMDV, and the overlapping images revealed an orange color, while those of the control group presented a yellow–green colour (Figure 4B). We continued to investigate the effect of HK2 on FMDV-induced autophagy in PK-15 cells by monitoring the number of endogenous LC3 dot clusters and the change in fluorescence of mRFP-eGFP-LC3. As shown in Figures 4C and D, HK2 further increased the number of endogenous LC3 dot clusters and enhanced the red fluorescence of mRFP-EGFP-LC3 in FMDV-infected PK-15 cells. These results suggested that HK2 could promote FMDV-induced autophagic flux. In addition, we analysed whether HK2-induced autophagy affects viral replication. First, HK2 overexpression increased the VP1 protein expression and 3D^pol^ mRNA level of FMDV in PK-15 cells (Figure 4E and F). After treatment with the autophagy inhibitors bafilomycin A1 and chloroquine (CQ), the VP1 protein expression and 3D^pol^ mRNA level of FMDV in HK2-overexpressing cells were significantly reduced (Figures 4E and F). These results indicated that HK2 promoted FMDV replication through autophagy.

**Fig. 5:**
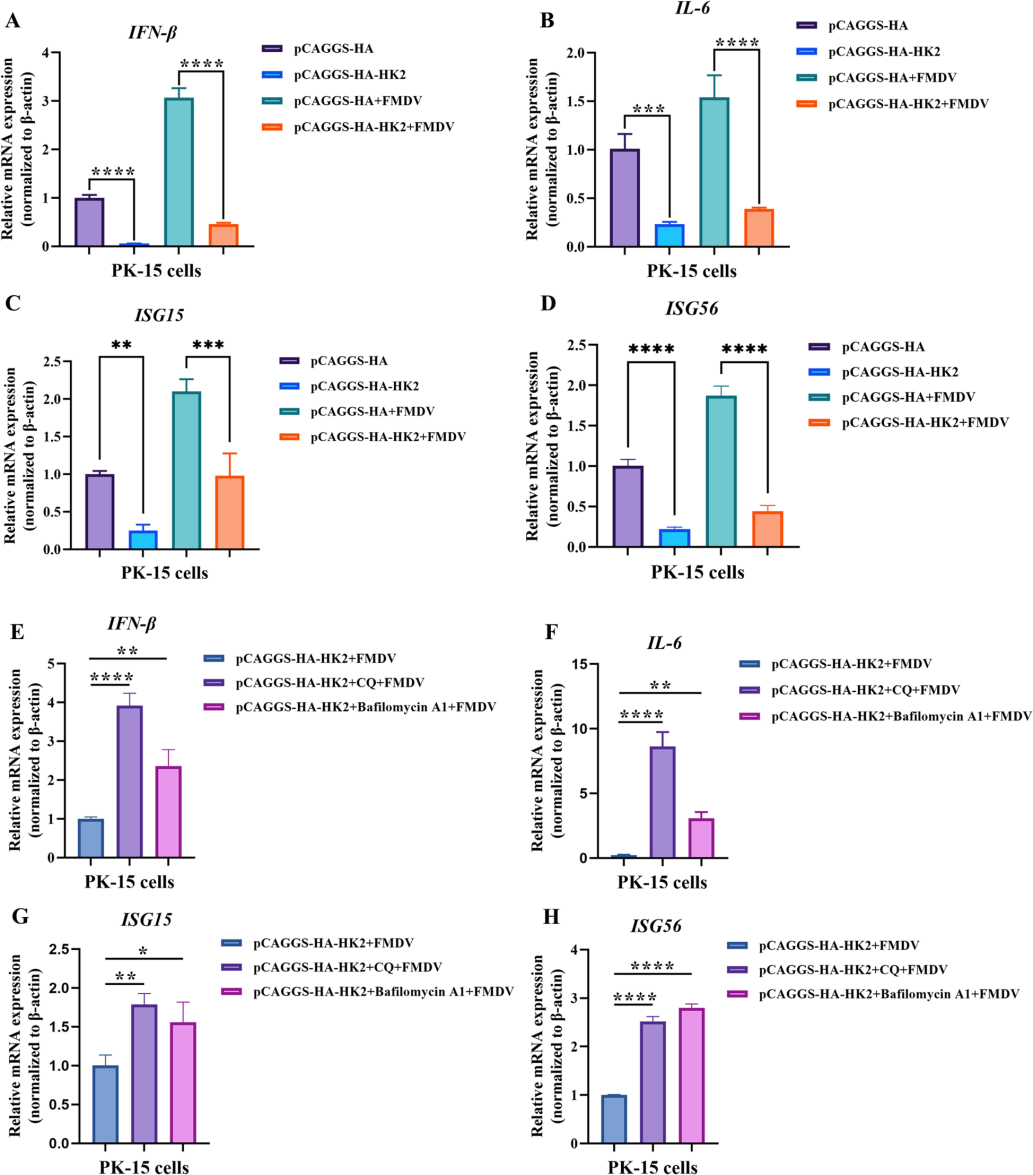
HK2 promoted FMDV replication by suppressing innate cellular immunity through autophagy. **A**–**D** PK-15 cells overexpressing pCAGGS-HA or pCAGGS-HA-HK2 were infected with or not infected with FMDV for 12 h. The mRNA levels of IFN-β, IL-6, ISG15 and ISG56 in the cells were measured by qRT-PCR. **E**–**H** PK-15 cells overexpressing pCAGGS-HA or pCAGGS-HA-HK2 were treated with bafilomycin A1 and CQ for 4 h, followed by FMDV infection for 12 h. The mRNA levels of IFN-β, IL-6, ISG15 and ISG56 in the cells were measured via qRT‒PCR. The error bars indicate the mean (± SD) of 3 independent experiments. *, *P* < 0.05; **, *P* < 0.01; and ***, *P* < 0.001 (one-way ANOVA).

To explore the effects of HK2 on the innate immune responses mediated by FMDV, PK-15 cells were transfected with pCAGGS-HA-HK2 or pCAGGS-HA for 24 h and then infected or not infected with FMDV for 12 h. Afterwards, the mRNA levels of IFN-β, IL-6, ISG15 and ISG56 in the cells were measured via qRT‒PCR. As shown in Figs. 5A–D, HK2 overexpression significantly inhibited the transcriptional activities of IFN-β, IL-6, ISG15 and ISG56, regardless of whether the cells were uninfected or infected with FMDV. Next, the autophagy inhibitors bafilomycin A1 and CQ were utilized to investigate whether HK2-induced autophagy affects FMDV-mediated innate immune responses. The results revealed that both bafilomycin A1 and CQ alleviated the inhibitory effect of HK2 on cytokines, including those in FMDV-infected PK-15 cells (Figures. 5E–H). Taken together, these results indicate that HK2 can enhance FMDV replication by suppressing innate cellular immunity through autophagy.

### HK2 interacted with IRF3/IRF7 and inhibited their expression

**Fig. 6:**
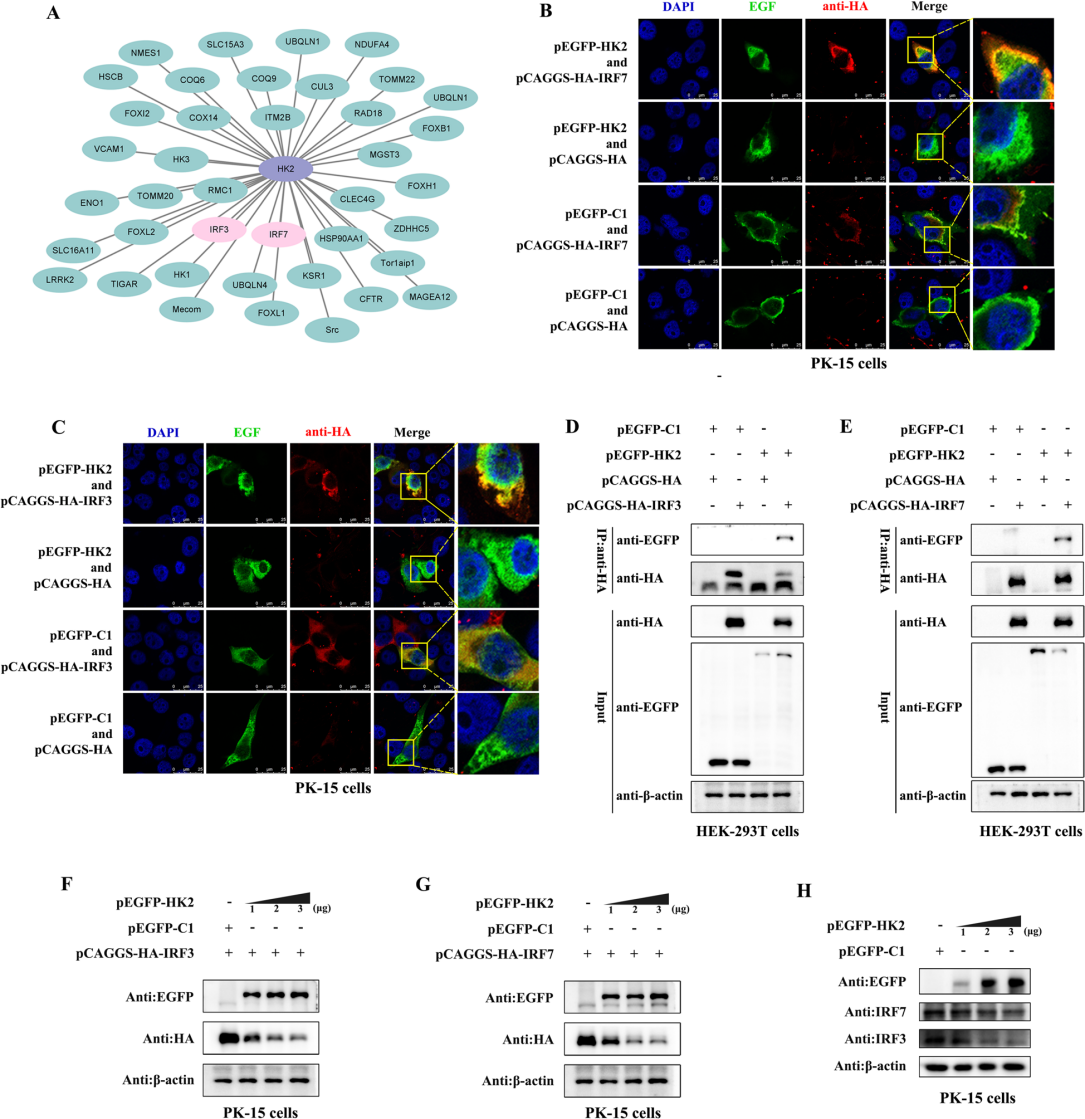
**HK2 interacted with IRF3 and IRF7 and inhibited their expression**. **A** The targets closely related to HK2 were predicted by BioGRID database. **B** and **C** PK-15 cells were co-transfected with pEGFP-HK2 and pCAGGS-HA-IRF3/IRF7, pEGFP-HK2 and pCAGGS-HA, pEGFP-C1 and pCAGGS-HA-IRF3/IRF7 or pEGFP-C1 and pCAGGS-HA for 24 h, fixed, stained with DAPI (blue) and antibodies against GFP (green) and HA (red), and then examined by confocal microscopy. **D** and **E** HEK-293T cells were co-transfected with pEGFP-HK2 and pCAGGS-HA-IRF3/IRF7, pEGFP-HK2 and pCAGGS-HA, pEGFP-C1 and pCAGGS-HA-IRF3/IRF7 or pEGFP-C1 and pCAGGS-HA for 24 h. The bound proteins were eluted and detected by western blotting with anti-HA and anti-GFP monoclonal antibodies. (F and G) 0 μg, 1 μg, 2 μg and 3 μg of pEGFP-HK2 were transfected into PK-15 cells together with 1 μg of pCAGGS-HA-IRF3/IRF7. Western blotting was used to evaluate the protein expression of GFP, HA and β-actin. (H) 0 μg, 1 μg, 2 μg and 3 μg of pEGFP-HK2 were transfected into PK-15 cells, and the protein expression of GFP, IRF3, IRF7, and β-actin was detected by western blotting.

The results of the present study revealed that HK2 antagonizes innate immunity to increase FMDV replication. To further explore how HK2 mediates innate immunity to regulate viral replication, the BioGRID database was first used to predict the key immune molecules closely related to HK2. Multiple potential interacting targets of HK2 were screened. Interestingly, interferon regulatory factor 3 (IRF3) and interferon regulatory factor 7 (IRF7) were also recognized as potential interacting partners of HK2 in the database (Figure 6A). IRF3 and IRF7 are important transcription factors that are required for type I interferon (IFN-α/β) production and the antiviral immune response [29, 30]. We further utilized confocal microscopy and co-IP to verify the relationship between HK2 and IRF3/IRF7. The confocal microscopy results revealed that HK2 and IRF3/IRF7 were colocalized in PK-15 cells (Figures 6B and C). Furthermore, the co-IP results revealed that HK2 coprecipitated with IRF3/IRF7 in HEK-293T cells (Figures 6D and E). In addition, the influence of HK2 on IRF3/IRF7 was also analysed, and the results revealed that HK2 inhibited both endogenous and exogenous expression of the IRF3/IRF7 protein in a dose-dependent manner (Figures. 6F-H).

### HK2 promoted IRF3/IRF7 degradation via an autophagy‒lysosome-dependent pathway

**Fig. 7:**
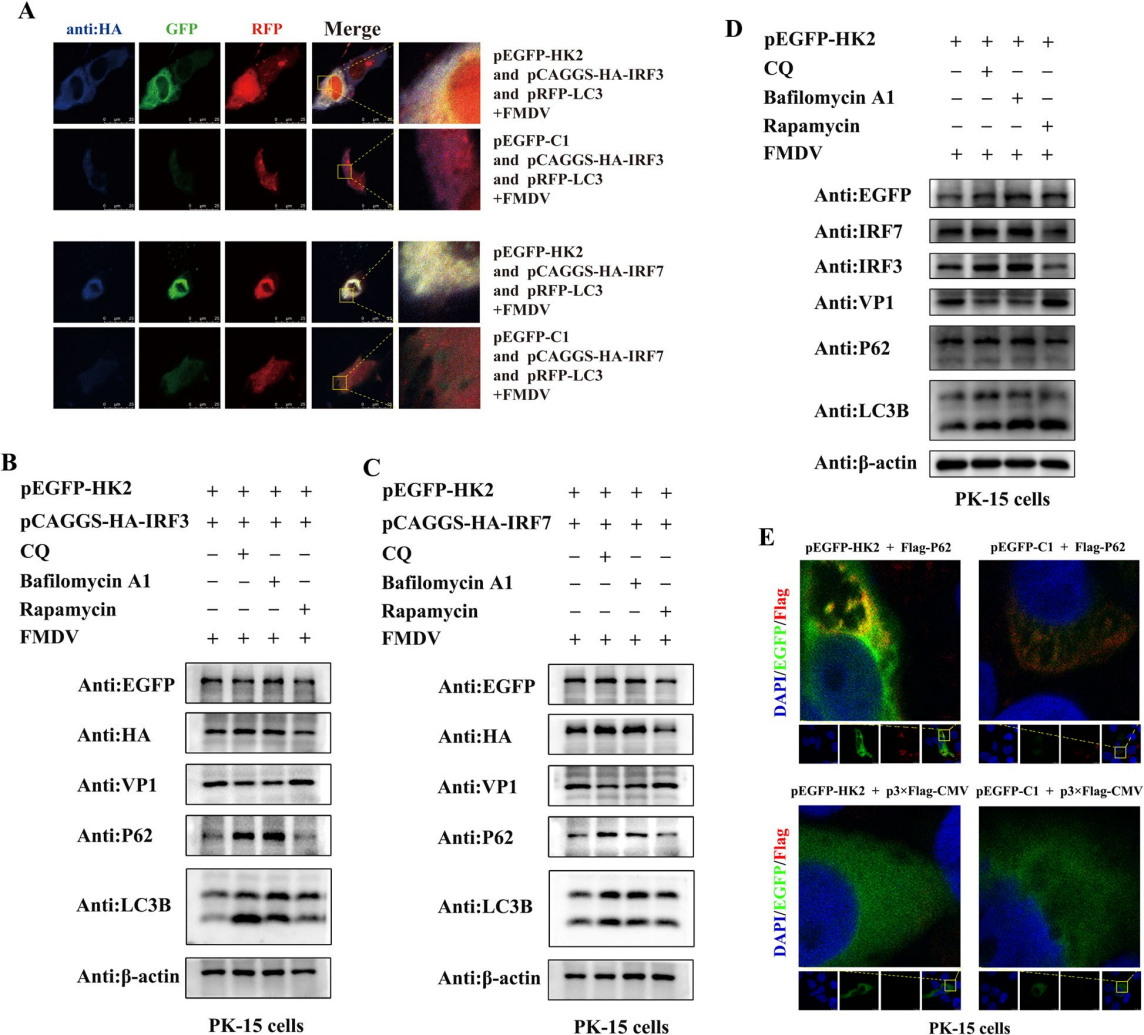
**HK2 utilizes autophagy to degrade IRF3/IRF7 during FMDV infection**. **A** PK-15 cells were co-transfected with pEGFP-HK2 and pCAGGS-HA-IRF3/IRF7 and pRFP-LC3 or with pEGFP-C1 and pCAGGS-HA-IRF3/IRF7 and pRFP-LC3 for 24 h, infected with FMDV for 12 h, fixed, stained with antibodies against HA (blue), GFP (green) and RFP (red), and then examined by confocal microscopy. **B** and **C** PK-15 cells were pretreated with the autophagy activator rapamycin (100 nM) or the inhibitors CQ (20 μM) and bafilomycin A1 (0.4 μM) for 4 h and then co-transfected with pEGFP-HK2 and pCAGGS-HA-IRF3/IRF7 for 24 h, followed by FMDV infection for 12 h. Western blotting was used to evaluate the protein expression of GFP, HA, VP1, p62, LC3B and β-actin. **D** PK-15 cells were transfected with pEGFP-HK2 after 4 h of pretreatment with the autophagy activator rapamycin (100 nM) or the inhibitors CQ (20 μM) and bafilomycin A1 (0.4 μM). The protein expression of GFP, IRF3, IRF7, VP1, p62, LC3B and β-actin was detected by western blotting. **E** PK-15 cells were co-transfected with pEGFP-HK2 and Flag-p62, EGFP-C1 and Flag-p62, EGFP-HK2 and p3 × Flag-CMV, or EGFP-C1 and p3 × Flag-CMV for 24 h, fixed, stained with DAPI (blue) and antibodies against GFP (green) and Flag (red), and then examined by confocal microscopy.

The above studies indicate that HK2 can mediate autophagy to antagonize the IFN response, thereby enhancing FMDV replication. Moreover, HK2 inhibited IRF3/IRF7 expression. These results suggest that HK2-induced autophagy is closely related to IRF3/IRF7 degradation during viral infection. HK2 may degrade IRF3/IRF7 through an autophagy‒lysosome-dependent pathway. To verify this hypothesis, the localization of HK2, IRF3/IRF7 and the autophagosome marker protein LC3 was observed via confocal microscopy. Figure 7A shows that LC3 closely colocalized with HK2 and IRF3/IRF7. Moreover, PK-15 cells were pretreated with an autophagy activator (rapamycin) or inhibitor (CQ or bafilomycin A1), co-transfected with pEGFP-HK2 and pCAGGS-HA-IRF3/IRF7, or infected with FMDV in sequence. The expression of IRF3/IRF7 and FMDV VP1 was tested by western blotting. As shown in Figures. 7B–D, the autophagy inhibitors CQ and bafilomycin A1 promoted both the endogenous and exogenous expression of the IRF3/IRF7 protein but inhibited the expression of FMDV VP1, in contrast to that in the control group. Conversely, rapamycin treatment exacerbated the degradation of IRF3/IRF7 and P62 and the expression of FMDV VP1 in PK-15 cells. These results demonstrated that HK2 mediated the degradation of IRF3/IRF7 through an autophagy‒lysosome-dependent pathway during FMDV infection. In addition, cytoplasmic colocalization of p62 and HK2 was detected via confocal immunofluorescence microscopy, suggesting that HK2 may degrade IRF3 and IRF7 through selective autophagy targeting mediated by the autophagy receptor p62 (Figure 7E).

## Discussion

Cellular metabolism is a series of processes by which an organism sustains itself [31]. As strict intracellular parasites, viruses can hold the metabolic pathways and metabolic enzymes of the host cell to create an environment conducive to their own replication [32, 33]. Glycolysis is the first metabolic pathway to be elucidated, and many studies have shown that glycolysis and its key enzyme HK2 play important roles in viral infection [8, 34]. PRRSV infection promotes glycolysis and the production of lactic acid, which targets MAVS to inhibit RLR signalling and thus promote viral replication [35]. The NS5A protein of HCV interacts with HK2 to accelerate glycolysis [34]. In this study, we observed that FMDV infection increased glycolysis and HK2 expression in PK15 cells. Moreover, HK2 promoted glycolysis in FMDV-infected cells and facilitated viral replication. These results suggest that FMDV, like most other viruses, can regulate host glycolysis. HK2 is a key molecule through which FMDV uses glycolysis to regulate its own proliferation. FMDV holds the host protein and regulates its function in favour of viral replication. In this study, the interaction between HK2 and FMDV VP3 was also explored, and HK2 increased VP3 expression in a dose-dependent manner. The mutual regulation of HK2 and VP3 and their effects on cellular glycolysis and viral replication need further analysis.

Various viruses have been reported to induce autophagy in host cells. As an important part of the cellular defense and protection mechanism, autophagy plays a vital role in resisting the invasion of pathogenic microorganisms. Coregulation between viruses and autophagy has given viruses multiple strategies to disrupt or exploit autophagy and obstruct cellular antiviral responses [36–38]. FMDV is known to induce autophagy. FMDV and viral proteins mediate autophagy to negatively regulate the innate immune response to maintain autoreplication [18, 35]. Interestingly, several controversial studies have reported that FMDV-induced autophagy interferes with virus development [39–41]. The relationship between FMDV and autophagy needs further investigation. In addition to glycolytic enzyme activity, HK2 also participates in the progression of autophagy and manages the innate immunity caused by viral infection through autophagy [15]. Multiple studies have shown that HK2 is closely related to autophagy [42]. HK2 promoted autophagy in response to glucose deprivation to protect cardiomyocytes [17]. Hypoxia-induced HK2 can enhance antiapoptotic effects in multiple myeloma via autophagy activation [43]. In this study, FMDV infection induced autophagy and promoted glycolysis and HK2 expression. These findings suggest a close association between FMDV and HK2 and autophagy. We further explored this relationship and discovered that HK2 enhanced autophagic flux during FMDV infection. Furthermore, HK2 reduced the production of interferon-associated cytokines (IFN-β, IL-6, ISG56, and ISG15) by promoting autophagy and subsequently promoted FMDV proliferation. Further analysis revealed that HK2 interacted with the interferon signalling pathway protein IRF3/IRF7 and degraded it via an autophagy‒lysosome-dependent pathway. A previous study revealed that HK2 can be recognized by the autophagy receptor p62 and that the protein expression of HK2 is positively correlated with that of p62 [44]. The findings concerning the colocalization of p62 and HK2 suggested that HK2 may degrade IRF3 and IRF7 through p62-mediated autophagy.

In conclusion, this study investigated the common regulation between glycolytic metabolism and FMDV and revealed that FMDV induced the glycolytic process in PK-15 cells. In addition, HK2 mediated the degradation of IRF3/IRF7 via an autophagy‒lysosome-dependent pathway to antagonize the interferon signalling pathway, thereby promoting viral replication. The findings of this study provide new insights into FMDV infection and the relationship between glycolysis and innate immunity. Glycolysis and its metabolic enzyme HK2 can create favourable environments for FMDV replication.

## Data Availability

The data that support the findings of this study can be found at “https://doi.org/10.6084/m9.figshare.27100819.v1”.
